# S-index periodicity detection based on multiple random spectral observations

**DOI:** 10.1038/s41598-023-48590-8

**Published:** 2023-11-30

**Authors:** Yu-Fu Shen

**Affiliations:** Changchun Observatory, National Astronomical Observatories, Chinese Academy of Sciences, Jingyuetan National Scenic Area, Changchun, 130117 China

**Keywords:** Stars, Stellar evolution

## Abstract

The solar magnetic activity cycle has a profound impact on our lives, yet its underlying causes remain elusive. While similar cycles have been observed in other stars, these results are scarce due to the significant challenge posed by the length of time required for observation. Continuous observation over an extended period is a luxury, making it difficult to gather large samples. However, multiple random observations can be made at a lower cost, and flexible time management enables non-professionals to become competent. In this study, we analyzed multiple random observations of the S-indices of several stars captured incidentally by the Large Sky Area Multi-Object Fiber Spectroscopic Telescope. Our findings reveal potential periodicity that could be associated with magnetic activity cycles. Notably, obtaining S-index is relatively less challenging, indicating the feasibility of developing dedicated commercial or charitable equipment for non-astronomers. A more extensive data is essential to establish a definitive correlation between S-index periodicity and magnetic activity cycles in stars, as well as to uncover additional magnetic activity cycles.

## Introduction

The S-index can be used to estimate the magnetic activity of stars. In this work, we have identified S-index periodicity, which appear to be connected to the magnetic activity cycle of stars. However, a comprehensive understanding of this relationship necessitates further observational data. The solar cycle represents a well-defined example of a magnetic activity cycle. The luminosity of the sun exhibits an 11-year cycle, and its number and area of sunspots follow similar patterns. It is widely accepted that these alternating changes are primarily attributed to periodic fluctuations in magnetic activity. The significance of studying the magnetic activity cycle in stellar physics cannot be overstated, and the influence that the magnetic activity cycle of stars has on planets always exceed expectations. The influence of the solar 11-year activity cycle on Earth’s climate is undeniable^1^. Climate change not only impacts the rise and fall of civilization, but also has a more profound effect on biological evolution. Maybe only stars with significant changes in magnetic activity can guide life towards a smarter direction, as the intelligence is the best way to face changing environments, and some deep-sea organisms far from changes have not evolved for billions of years. This implies that long-term changes in stellar magnetic activity may have an impact on the Drake equation’s fi term^2^. However, the current understanding of the magnetic activity cycle of stars is far from satisfactory. While magnetic activity cycle candidates have also been identified in other stars^3–12^, the majority can only be regarded as candidates due to the limited availability of long-term observational data for stars. Consequently, the current numbers and accuracy of stellar activity cycle samples are inadequate, making it difficult to conduct comprehensive statistical research, for example, the cycle sequence^13^.

Long-term observations are always expensive and difficult to apply to a large number of targets. Great works contains tens of years of Ca  ii H and K observations such as the Mount Wilson HK project^14^ are hard to replicate. Even Kepler’s outstanding task of continuously observing approximately 200,000 targets for four years^15,16^ is still insufficient for statistical research on magnetic activity cycles. Compared to photometric surveys (such as Gaia^17^, $\sim$1.5 billion stars), 200,000 is negligible; as for the observation time, if the typical magnetic activity cycle is around 11 years, 4 years are still too short. The existence of an 11-year cycle can be detected by 4-year long data, but the specific length of the cycle cannot be determined^18^. The study of stellar magnetic activity cycles calls for a new, low-cost observation mode, and the Large Sky Area Multi-Object Fiber Spectroscopic Telescope (LAMOST^19^) provides inspiration.

The fiber spectroscopic telescopes like LAMOST require setting the coordinates of the target before observation, which relies on input catalogs. However, due to the lack of supporting photometric surveys, the development of input catalogs for LAMOST is more complex, and repetitive observations are inevitable. Out of the 7,181,840 stars with Gaia DR3 id in the LAMOST DR8 low resolution spectrum, 87,818 have been observed 5 or more times, with time spans, the difference between the beginning and the end of observations, ranging from 0 (observed multiple times in a day) to 8.4 years, with 80% being over two years. The S-index based on Ca  iiH K line is an effective indicator for detecting the magnetic activity cycle of stars. To avoid misunderstandings, it should be noted that the 11-year activity cycle of the sun is actually the total irradiance cycle, spot number cycle, etc. We know that S-index, total irradiance, spots, flares, etc. are all related to magnetic activity^20,21^. Besides, the spots rotate with the star, causing rotational modulations^22^, and the amplitude of the light curve with rotational modulations is related to the total area of spots, then $R_{var}$ is defined, which is related to the amplitude, so there are $R_{var}$ cycles^9^. Therefore, various characteristics can be chosen for periodicity analysis. Nevertheless, synchronization among these cycles remains undetermined. Interference from prolonged gigantic spot evolution and occasional flares represents known challenges, as they have the potential to impact both the light curve and the S-index.

This study has considered 87,818 stars that have been observed 5 or more times and discovered that 6,196 of them have S-index periodicity with false alarm probabilities (FAP) < 0.05 (3946 with FAP < 0.01; 11,348 with FAP < 0.32). Due to the unique characteristics of large time spans and uneven time distribution of data, this study specifically designed Fourier analysis method and method for calculating FAP. To confidently conclude that the periodicity is related to magnetic activity cycles, more data must be obtained. However, expecting to continually mine targets that have been observed multiple times in various spectroscopic surveys as a long-term solution is not feasible, as spectroscopic surveys generally do not wish to repeatedly observe the same targets. Compared to the samples obtained from photometric surveys, the spectroscopic survey still differs by about two orders of magnitude. Therefore, it is crucial for the spectroscopic survey to obtain more spectra of celestial bodies. LAMOST is also working on revising the catalog, attempting to reduce the probability of repeating observations of previously observed celestial bodies. We believe that a spectroscopic survey is not necessary in this situation and that involving more people can be a better approach.

The bands required to calculate the S-index are quite narrow ($\sim 100 \mathring{\mathrm A}$), necessitating low spectral resolution and relatively low signal-to-noise ratios, which makes observing at a reasonable cost feasible. If automated devices dedicated solely to observing S-index were produced in large quantities in collaboration with companies that sell personal telescopes, the cost could be significantly reduced. Low-cost, intelligent, and compact observation equipment could be contributed to various collaborators who might provide a field station, such as meteorological departments or geological departments. Residents live in top floors, farmers who often reside in areas with low nighttime sky-light backgrounds, and anyone interested in scientific activities who is willing to go out and find a suitable location for observing when they have free time could all be potential partners. Even if the willingness of these individuals to purchase the equipment is limited, and the project proves unprofitable, utilizing the strategy of renting equipment and providing subsidies based on the quantity and quality of data collected could result in lower costs than using a professional telescope. For individuals residing in poor areas where observing conditions are typically better, the program’s subsidies can be charitable. By participating in observations and providing data, these individuals would also have a reason to become co-authors of the paper, which could be highly motivating and have a long-term impact on scientific popularization.

## Methods

If a target is only observed two or three times, conducting periodic analysis on it is meaningless. Due to the unknown frequency and phase of the signal, it is difficult to derive a meaningful minimum number of observations. This work randomly generated a sine curve with a length of 1 and a period of 1.2 containing Gaussian noise, and then randomly selected n points to observe whether the power spectrum of these n points showed a peak at about 1.2. This rough method found that the success rate is about 25% for 4 data points, 80% for 5 data points, and 96% for 6 data points. Therefore, we did not consider targets that were observed less than 5 times. However, there is still a difference between the observation mode of LAMOST and completely random, as some targets are densely observed multiple times within a few days, and the contribution of these observations is actually only equivalent to once. Therefore, this work is based on a 30-day unit. If there are multiple observations within 30 days, the weighted mean value ($weight=1/error^2$) within 30 days is considered as a single observation, and the total error of propagation is used as the error range. In addition, there is no analysis of observations with a time span of less than 2 years. Finally, 13,660 stars are analysed.

This work employs S-index^23,24^, the emission in the Ca  ii H and K lines relative to the continuum, as the primary metric to gauge the intensity of magnetic activity,

1 $$\begin{aligned} S=8 \alpha \cdot \frac{H+K}{R+V} \end{aligned}$$

where *H* and *K* are the fluxes integrated in 1.09 $\mathring{\mathrm A}$ FWHM triangular windows centered on the line cores of 3968 and 3934 $\mathring{\mathrm A}$. *R* and *V* are the fluxes integrated in 20 $\mathring{\mathrm A}$ rectangular windows centered on 4001 $\mathring{\mathrm A}$ and 3901 $\mathring{\mathrm A}$. $\alpha = 1.8$ is the normalization number^14^. The inverse variances of the spectra provided by LAMOST are used to calculate the error range of S-indices.

It is important to recognize that the periodic signal obtained by directly applying the Discrete Fourier Transform (DFT) to randomly distributed data points in time is not reliable. This is due to the fact that there are instances where the frequency with the highest power is non-physical. If this frequency is used to fit the data points, it can lead to a situation where the minimum value of the S-index is less than 0, which contradicts the definition of the S-index. To address this issue, the method employed in this work is to fit the data points using a sine curve, which is inherently consistent with the DFT. This could avoid being misled by non-physical but stronger signals. This approach requires additional constraints on parameters such as amplitude to ensure that the S-index remains non-negative over a cycle. Non-linear least squares fitting is used, where $weight=1/error^2$. In

2 $$\begin{aligned} f=a sin (\frac{2 \pi }{T} x + c)+d \end{aligned}$$

where 2 observation time span/numbers of observation < T < 2 observation time span; 0 < a < 0.5 (max(S) - min(S)); average(S) - 0.5 (max(S) - min(S)) < d < average(S) + 0.5 (max(S) - min(S)). The lower limit of *T* is set to prevent data fitting with excessively high frequencies; the upper limit of *T* is set due to the decreased reliability of cycles much longer than the observation time span. Some discussions about the cycles longer than observation time span are given in Sect. 3. These settings are not fixed and are not the most ideal, but they can achieve the goal. The periodic signal obtained through this operation provides a reliable method for calculating the FAP. If only the frequency with the strongest power is analyzed, it is likely to obtain a very low FAP, but the result is nonphysical. The method for calculating the FAP in this work is to obtain the fitting curve *f* and calculate the sum of residual squares ($\sigma _0^2$) of the observed S-index sequence and *f*. Afterwards, keeping the time distribution of the S-index sequence unchanged, randomly shuffling the values of the S-index and calculating a new sigma, perform this operation 100 times, where the rate of $\sigma > \sigma _0$ is the FAP. This method of calculating the FAP eliminates false cycles caused solely by the periodic distribution of observation.

It should be noted that using a sine function for fitting may not be the best approach, as the variation of stellar magnetic activity does not always follow the pattern of a sine function. Typically, the magnetic activity strengthens rapidly and then decays slowly, exhibiting asymmetry^25^. Unfortunately, this characteristic is difficult to verify when the data points are too sparse. However, considering that the magnetic activity cycle itself is a quasi-periodic, and there are differences between different solar maximum years, it is not meaningful to use more complex functions for fitting, unless future research leads to a deeper understanding of stellar magnetic activity and more accurate modeling can be conducted. Nonetheless, any function can actually be decomposed into multiple sine functions. If in the future, other forms of functions are found to better fit stellar magnetic activity, this will imply that the 11-year cycle similar to that of the Sun is just an illusion, and in reality, it is driven by multiple magnetic activity factors with different frequencies.

## Discussion

**Figure 1:**
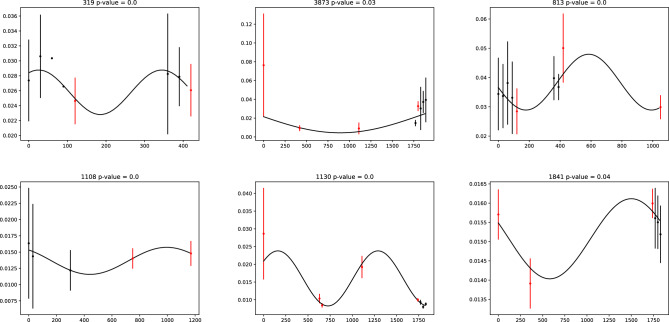
From left to right, top to bottom, the first panel displays Gaia DR3 3423349763984800000, the second panel displays Gaia DR3 3373123729231227776, the third panel displays Gaia DR3 3424636604905437696, the forth panel displays Gaia DR3 1001300195463577344, the fifth panel displays Gaia DR3 65270416136306560, the sixth panel displays Gaia DR3 598382888153108992. The cycle period and FAP are displayed at the top of each panel. The curve represents the sine fitting of the S-index periodicity. Red points means the star has only been observed once by LAMOST within the corresponding 30-day.

**Figure 2:**
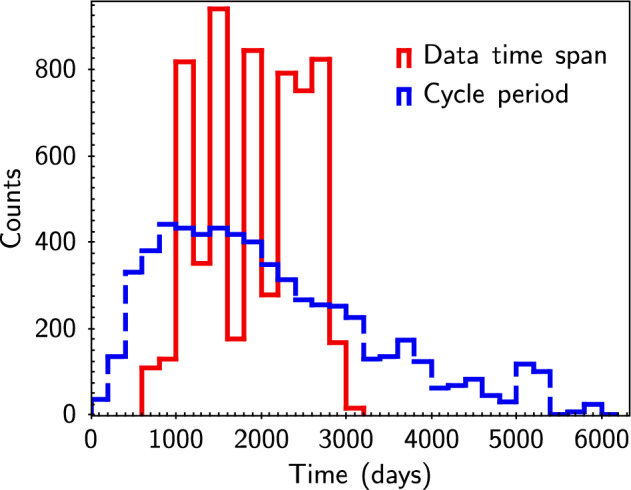
Histogram over time of S-index periodicity and time span of analyzed sample.The red solid line represents the histogram of time span for 13,660 stars, while the blue dashed line depicts the histogram of S-index periodicity for 6196 stars.

**Figure 3:**
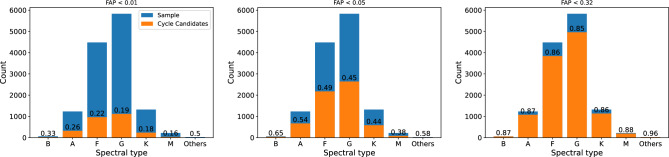
Histogram over spectral types of S-index periodicity and analyzed sample. For all three panels, the blue bars represent the various spectral types present in the analyzed sample, while the orange bars represent the various spectral types in the cycle candidates. The numbers indicate the proportion of cycle candidates among each spectral type. “Others” refer to special celestial bodies such as white dwarfs and binary stars.

This study has identified 6196 stars with S-index periodicity (FAP < 0.05), constituting 45% of the total (83% if FAP < 0.32; 29% if FAP < 0.01). Figure 1 provides several examples. The distribution of S-index periodicity and the time span distribution of sample are presented in Figure 2; No significant correlation was found between the two, indicating that the impact of the sampling function is relatively minimal. Based on the spectral classification provided by LAMOST, the distribution and proportion of samples and samples with S-index periodicity are shown in Fig. 3. The “others” in the figure represents targets classified as special stars such as white dwarfs, binary stars, and variable stars; their proportion is very small, so no more research has been conducted. Baliunas et al.^26^ reported that 60% of low main sequence stars with activity cycles were found. The results of this study were slightly lower, possibly due to the limited data time span and the loss of some long-period samples. Figure 3 also indicate that the proportion of stars with S-index periodicity increases with temperature, which is not a reliable conclusion, as magnetic activity fluctuations always become more pronounced with decreasing temperature. If the data points fluctuate too much, a higher frequency must be used for fitting. However, when the number of data points is limited, the result of high-frequency fitting is not reliable, and such fits will be excluded. It should be noted that some B-type and A-type stars have also been found to exhibit S-index periodicity, which is unexpected due to their distinct structure, and their surfaces lack convection, resulting in low magnetic activity. In fact, the S-index has a weaker correlation with magnetic activity in B-type and A-type stars, and may be more strongly affected by stellar winds. For example, stellar winds can lead to blueshifted absorption in the spectrum^27^. Therefore, the S-index periodicity found for B-type and A-type stars are more likely to be related to other physical phenomena. Additionally, the LAMOST spectral classification is automated and subject to potential classification errors, so the target may not actually be a B-type or A-type star.

In addition, there are other factors that could have led to false S-index periodicity in this work. Firstly, there are accidental phenomenon such as gigantic spot evolution and incidental flares which could affect the results. Secondly, the area of sunspots in the line of sight will vary with the rotation of the star. If the observations are made when the sunspots are concentrated on the back or front, the magnetic activity of the star can be underestimated or overestimated. If multiple observations are taken over a short period of time and averaged, the effect of this can be reduced. Therefore, referring to Fig. 1, the results obtained based on data points with black error ranges will be more reliable. Thirdly, the relation between stellar magnetism and S-index is still unclear, for example, Sowmya et al.^28^ pointed out that the S-index maybe affected by the inclination angle of the stellar rotation axis. This indicates that if a star exhibits significant nutation, an S-index periodicity should arise, which could potentially introduce a spurious S-index periodicity, but also provides the possibility for studying more complex motions of the star.

**Figure 4:**
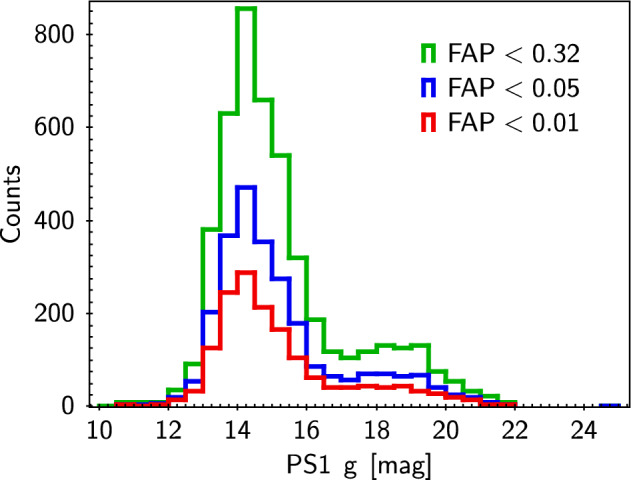
Pan-STARRS1 g band distribution of stars with S-index periodicity in this work.

Finally, here is a rough discussion on the possibility of non-professional astronomers detecting S-index. The distribution of g-band magnitudes (Pan-STARRS1 g band^29^) for sample stars discovered with S-index periodicity is shown in Fig. 4. The g-band magnitude is close to the wavelength required for the S-index. The maximum aperture of a telescope that an individual can afford is up to about 30cm, and the light-gathering ability is about 0.6% of that of the LAMOST 4m aperture, with a difference of about 5.5 magnitudes. Thus, a non-professional astronomer can observe stars with magnitudes around 15 or 16, covering a large number of target stars. If we cooperate with non-astronomy professionals who can provide an observation station, it is possible to further expand the aperture of telescopes. However, achieving such observation depth requires long or multiple exposures, the total exposure time must reach 5400 seconds, then the signal-to-noise ratio of the spectrum near Ca  ii H and K can reach 1.5, if the spectral resolution $R=1800$. If the spectral resolution is reduced, it can shorten the exposure time. A long-time explosion means that equipment leveling is required before each observation, which in turn requires the observer to have certain experience.

## Data Availability

The data analysis was performed with custom Python scripts following the standard procedure. The code is available on request from shenyf@cho.ac.cn. The spectra of LAMOST DR8 have been released internationally (https://www.lamost.org/dr8/v2.0/).
